# Discovering predisposing genes for hereditary breast cancer using deep learning

**DOI:** 10.1093/bib/bbae346

**Published:** 2024-07-22

**Authors:** Gal Passi, Sari Lieberman, Fouad Zahdeh, Omer Murik, Paul Renbaum, Rachel Beeri, Michal Linial, Dalit May, Ephrat Levy-Lahad, Dina Schneidman-Duhovny

**Affiliations:** The Rachel and Selim Benin School of Computer Science and Engineering, The Hebrew University of Jerusalem, Jerusalem 9190401, Israel; The Fuld Family Medical Genetics Institute, Shaare Zedek Medical Center 12 Bayit St., Jerusalem 9103101, Israel; The Eisenberg R&D Authority, Shaare Zedek Medical Center, 12 Bayit St., Jerusalem 9103101, Israel; Faculty of Medicine, The Hebrew University of Jerusalem, Ein Kerem PO Box 12271 Jerusalem 9112102, Israel; The Fuld Family Medical Genetics Institute, Shaare Zedek Medical Center 12 Bayit St., Jerusalem 9103101, Israel; The Eisenberg R&D Authority, Shaare Zedek Medical Center, 12 Bayit St., Jerusalem 9103101, Israel; The Fuld Family Medical Genetics Institute, Shaare Zedek Medical Center 12 Bayit St., Jerusalem 9103101, Israel; The Eisenberg R&D Authority, Shaare Zedek Medical Center, 12 Bayit St., Jerusalem 9103101, Israel; The Fuld Family Medical Genetics Institute, Shaare Zedek Medical Center 12 Bayit St., Jerusalem 9103101, Israel; The Eisenberg R&D Authority, Shaare Zedek Medical Center, 12 Bayit St., Jerusalem 9103101, Israel; The Fuld Family Medical Genetics Institute, Shaare Zedek Medical Center 12 Bayit St., Jerusalem 9103101, Israel; The Eisenberg R&D Authority, Shaare Zedek Medical Center, 12 Bayit St., Jerusalem 9103101, Israel; Department of Biological Chemistry, Institute of Life Sciences, The Hebrew University of Jerusalem, Edmond J. Safra Campus, Givat Ram, Jerusalem 91904, Israel; The Fuld Family Medical Genetics Institute, Shaare Zedek Medical Center 12 Bayit St., Jerusalem 9103101, Israel; The Eisenberg R&D Authority, Shaare Zedek Medical Center, 12 Bayit St., Jerusalem 9103101, Israel; Clalit Health Services, Jerusalem, Israel; The Fuld Family Medical Genetics Institute, Shaare Zedek Medical Center 12 Bayit St., Jerusalem 9103101, Israel; The Eisenberg R&D Authority, Shaare Zedek Medical Center, 12 Bayit St., Jerusalem 9103101, Israel; Faculty of Medicine, The Hebrew University of Jerusalem, Ein Kerem PO Box 12271 Jerusalem 9112102, Israel; The Rachel and Selim Benin School of Computer Science and Engineering, The Hebrew University of Jerusalem, Jerusalem 9190401, Israel

**Keywords:** hereditary breast cancer, family studies, missense variant analysis, peroxisome fatty acid metabolism, deep learning, structural biology

## Abstract

Breast cancer (BC) is the most common malignancy affecting Western women today. It is estimated that as many as 10% of BC cases can be attributed to germline variants. However, the genetic basis of the majority of familial BC cases has yet to be identified. Discovering predisposing genes contributing to familial BC is challenging due to their presumed rarity, low penetrance, and complex biological mechanisms. Here, we focused on an analysis of rare missense variants in a cohort of 12 families of Middle Eastern origins characterized by a high incidence of BC cases. We devised a novel, high-throughput, variant analysis pipeline adapted for family studies, which aims to analyze variants at the protein level by employing state-of-the-art machine learning models and three-dimensional protein structural analysis. Using our pipeline, we analyzed 1218 rare missense variants that are shared between affected family members and classified 80 genes as candidate pathogenic. Among these genes, we found significant functional enrichment in peroxisomal and mitochondrial biological pathways which segregated across seven families in the study and covered diverse ethnic groups. We present multiple evidence that peroxisomal and mitochondrial pathways play an important, yet underappreciated, role in both germline BC predisposition and BC survival.

## Introduction

Breast cancer (BC) is a prevalent disease among women globally. In Western countries, lifetime risk is ~13% and is affected by both genetic and environmental factors. The individual’s risk assessment primarily considers family history, clinical assessment, and screening tests. Germline pathogenic variants in cancer predisposition genes (CPGs) account for ~5%–10% of all BC cases. Over the last two decades, genetic factors for BC susceptibility have been identified. As a result, germline genetic testing has become a crucial part of clinical genetics practice, aiding in the prevention and treatment of BC in high-risk individuals. Among the well-established breast CPGs are BRCA1 and BRCA2, which are highly penetrant tumor suppressor genes; alterations in these genes account for ~30% of familial BC cases [1] and ~2% of all BC cases. Other genes with confirmed high lifetime risks include PALB2, PTEN, TP53, CDH1, and STK11 [2]. Over the years, the list of CPGs for BC was extended to also include BARD1, CHEK2, RAD51C, and RAD51D [3–5]. Collectively, these known genes can only explain ~6% of familial BC cases [6–9]. It is anticipated that there are additional familial cases involving highly or moderately penetrant rare pathogenic variants that cannot be detected in classical population studies. Furthermore, previous studies have been largely based on women of European origin and women of other ancestries and ethnic groups are not well represented in large population cohorts and are therefore overlooked.

A common approach to further expanding the BC catalog of inherited susceptibility genes relies on inspecting the genetic landscape of affected individuals. Genome-wide sequencing reveals multiple variants whose contribution to malignancy is difficult to estimate. Even when accounting only for variants shared between affected family members, there are yet hundreds of variants in genes with unknown relation to cancer [10]. Evaluating the pathogenicity of missense variants poses a significant challenge in human genetics in general. This is even more pronounced in the field of cancer predisposition where penetrance is age-dependent and incomplete. Although more common than null variants, most missense variants do not alter protein structure and function [11] and bioinformatic tools aiming at classifying their pathogenicity are not sufficiently accurate.

Recent advances in the field of machine learning (ML) have resulted in breakthroughs in predicting protein structure and function. Studies using various ML techniques showed state-of-the-art performance in predicting the pathogenicity of variants at DNA [12] and protein levels [13–16] matching the accuracy of deep mutational scans (DMSs). Additionally, recent breakthroughs in structure prediction, such as AlphaFold2 [17], significantly increased the structural coverage of the human proteome [18–20]. These new tools enable variant analysis at the protein level [21] opening the door for more robust studies of familial, rarely occurring variants [22], which would not be possible using classical population analysis. Additionally, structural analysis of deleterious variants might also provide clues for their mechanism at the protein level.

In this study, we seek to unveil new, overlooked variants and CPGs by analyzing a unique cohort of 12 families with evident familial predisposition to BC and negative for all known BC predisposition genes. All families in the study present a higher prevalence of BC and a younger age at diagnosis compared to the general population, indicating a high pretest likelihood of germline predisposing pathogenic variants. In addition, the participating families are of diverse origins, which are mostly not well represented in other studies. Whole genome sequencing was undertaken for all participants. We focus on a subset of 1218 missense variants that are very rare in the general population and shared between affected family members.

We develop an ML-based pipeline that ranks the pathogenicity of variants using scores obtained from three complementary state-of-the-art deep learning methods for variant effect prediction. Our ranking protocol seeks for consensus among the deep learning models. We focus on the top ranked variants that account for ~6% of all missense variants reviewed in this study. Further analysis revealed missense variants in eight genes segregating in seven different families, involved in various peroxisomal pathways. Peroxisomes are cellular organelles that play a key role in lipid metabolism, reactive oxygen species (ROS), calcium regulation [23–25], and fatty acid oxidation, which have all been shown to have an important role in cancer [26–28]. So far, these genes have not been identified as BC-related. Moreover, using the database of BC transcriptomes compiled by Kaplan–Meier (KM) plotter [29] and tumor samples from cBioPortal [30, 31], we find that alterations in these genes significantly affect BC survival. Our results highlight the importance of peroxisomal mechanisms in both germline predisposition to BC and in BC pathogenicity.

## Results

### Patients and variants

Cancer patients from 12 high-risk families were recruited for the study (Table 1, Fig. S6). All the patients tested negative previously for known cancer predisposition genes. The dataset included 40 subjects, 39 female and one male. Overall, there were 35 BC patients, a colon cancer patient, a pancreatic cancer patient, and 3 unaffected female family members, in families 5 (age 79), 9 (age 62), and 12 (age 62). The mean age for female BC was 48.4 (range: 26–70), and the mean youngest age for BC diagnosis across families was 38.9 (range: 26–47). All available sequencing data were analyzed—no filtration or selection were applied. Genome data from each patient were aligned to the reference genome. Only rare missense variants identified in at least two affected family members were included in our final analysis (Table S2 available online at http://bib.oxfordjournals.org/). The number of missense variants that were identified in each family varies considerably (Table 2). Our final dataset consists of 1218 missense variants in 1123 unique genes. Nine variants occurred in all affected members of two or more families.

**Table 1:** Information about the families in our dataset.

Family	Origin	Family history of breast cancer		Study participants^a^		
		Family members with breast cancer (*N*)	Youngest age of cancer diagnosis in the family (years)	Study participants (*N*)	Study participants affected with breast cancer	Age at breast cancer diagnosis^b^: mean (range)
**F1**	Persian-Iraqi-Kurdish	4	45	3	3	53 (45–67)
**F2**	Moroccan	4	26	1	1	26 (26)
**F3**	Persian	10	40	3	3	59.3 (40–70)
**F4**	Moroccan	12	36	5	5	41.2 (36–46)
**F5**	Yemen	3	44	3^**c**^	2	46.5 (44–49)
**F6**	Tunisian	7	44	3	3	55 (44–63)
**F7**	Moroccan	11	35	5	5	50.2 (46–57)
**F8**	Ashkenazi	3	36	3^d^	3	38 (36–40)
**F9**	Ashkenazi	3	34	3^**c**^	2	38.5 (34–43)
**F10**	Persian	4	47	3	3	55.3 (47–61)
**F11**	Persian	6	38	3^e^	2	48.5 (38–59)
**F12**	Persian	7	42	5^**c**^	3	50.67 (42–60)

a Study participants are those who underwent WGS. Family members who did not consent or had deceased were not included in the study and were not sequenced.

b If patients had multiple breast cancer diagnoses, the youngest age was counted.

c One participant is unaffected.

d One participant had colon cancer.

e One participant had pancreatic cancer.

**Table 2:** Number of candidate pathogenic variants per family stratified by scoring function.

Family	Total variants	Candidate pathogenic variants	DSRank only	FamRank only	DSRank and FamRank
**F1**	116	7	4	6	3
**F2**	9	2	0	2	0
**F3**	80	7	7	6	6
**F4**	44	4	4	4	4
**F5**	229	15	9	13	7
**F6**	122	8	3	7	2
**F7**	230	14	11	9	6
**F8**	20	2	0	2	0
**F9**	148	9	4	8	3
**F10**	243	17	14	11	8
**F11**	81	7	6	3	2
**F12**	129	7	3	7	3

Note some variants recur in multiple families.

### Variant analysis pipeline

**Figure 1:**
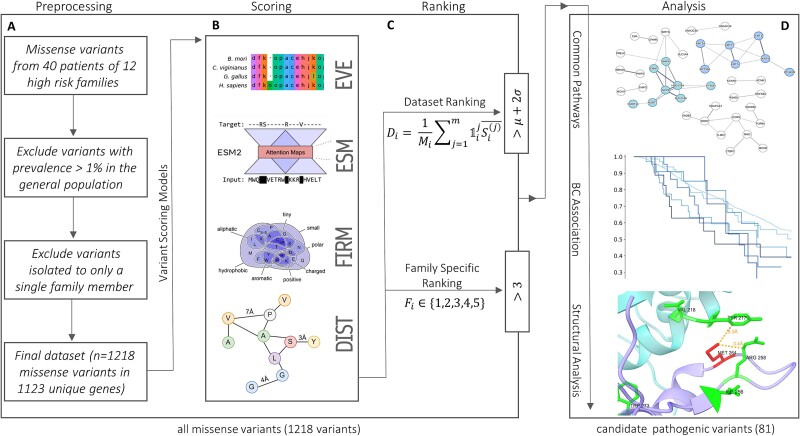
Variant analysis pipeline. (A) Preprocessing flow. (B) ML scoring EVE (EVEmodel), ESM (ESM-1v), FIRM (functional impact rating at the molecular level) and structural algorithm DIST. (C) $Di$ and $Fi$represent the dataset and family ranking of variant $i$, $m$ the number of ML models ${\mathbb{1}}_i^j$ indicator of whether variant $i$ was scored by model $j$, ${S}_i^{(j)}$ the variant normalized sore of model $j$ (min max normalization over entire dataset), ${M}_i$ is given by ${\sum}_{j=1}^m{\mathbb{1}}_i^j$, *μ* and *σ* are the mean and SD of DSRank over the entire dataset. (D) Analysis flow.

Our pipeline consists of three stages (Fig. 1). In the first stage (Scoring), each variant receives up to four scores from pathogenicity potential predictors. In the second stage (Ranking), the scores are normalized, and using two ranking algorithms, a subset of candidate pathogenic variants is assembled. In the third stage (Analysis), we look for common pathways and mechanisms among the candidate genes and evaluate them in context of BC.

#### Scoring

##### Machine learning–based scoring functions

We employ three ML models: EVEmodel [15], ESM-1v [13, 14], and FIRM [12] that were validated on variant effect prediction (VEP) benchmarks and demonstrated state-of-the-art performance (Table S3 available online at http://bib.oxfordjournals.org/). Each model was trained on a unique dataset and used a different approach to solve the VEP task. EVEmodel [15] is an evolutionary model that relies on multiple sequence alignment of the protein family. It is an unsupervised model that relies on the distribution of sequence variation across organisms for predicting variant pathogenicity. ESM-1v is a large protein language model (650M parameters) trained on 98M diverse protein sequences. Unlike EVEmodel, which is trained to predict variants in specific protein families, ESM-1v employs a general protein-centric approach without considering evolutionary information from multiple sequence alignments. To complement EVEmodel and ESM-1v, we also include FIRM (Functional Impact Rating at the Molecular-level)—an ML model based purely on molecular biochemical properties that are used to predict the functional impact of any genetic variants at the protein level. The models were validated on ClinVar [32] or using deep mutational scans assays (Table S3 available online at http://bib.oxfordjournals.org/).

**Figure 2:**
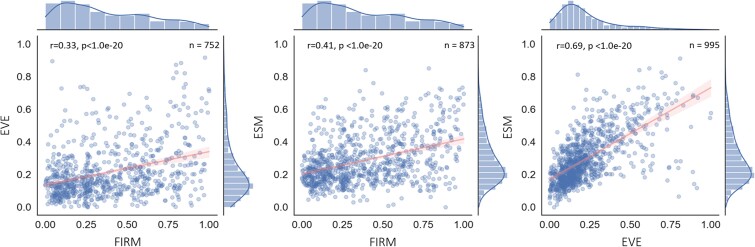
Pairwise model correlation. Main figures: pairwise models score over the entire dataset, side figures: scores distribution per model over the entire dataset. Regression line (red) with 95% confidence interval (pink shade), *r*—Pearson correlation coefficient, *P*—two-tailed *P*-value, *n*—number of samples.

Recent work by Jagota *et al*. [33] demonstrated that combining ESM-1v, EVE, protein structure, and amino acid representation resulted in improved performance over EVE and ESM-1v alone. We hypothesize that each model captures different features relevant for VEP and thus, employing committee-based classification algorithms using these models will improve results over classification based on a single model. To support this claim, we calculate pairwise correlations between the three scores and find that the ML models are partially positively correlated (Fig. 2). The correlation is higher between the sequence-based models EVEmodel and ESM-1v. In contrast, it is lower between FIRM and the sequence-based models. In essence, although models tend to agree on variants’ pathogenicity, it is likely that they also capture different features affecting the pathogenic potential that decorrelates them to some extent. This is most likely a consequence of the different approaches employed by the models. Recently, AlphaMissense [19], a new SOTA pathogenicity affect predictor, was released. Although it was not incorporated into our pipeline, it shows a significant correlation to all models used in our pipeline as well as to the aggregated ranking score DSRank (Fig. S8).

##### Protein–protein interaction DIST score

It is well established that deleterious variants in known cancer-related genes affect protein–protein interactions (PPIs) [34, 35]. To account for the potential effect of variants on PPIs, we also designed an additional DIST score that is based on available structural information. The DIST score is calculated by measuring the minimal distance between the variant’s atom positions and the closest atoms of the adjacent chains in all PDB structures containing the variant (Methods). Only 12% of variants in our dataset had available structures in the PDB for this analysis.

#### Ranking

There are a number of challenges with the interpretation of the pathogenicity scores produced by the scoring methods. First, the ML models do not provide a clear threshold for a variant to be considered pathogenic; rather, they output a numerical value that might be difficult to interpret out of context. Second, our dataset is inherently biased toward more pathogenic variants due to preprocessing that discards prevalent (>1%) variants in the general population [36]. Third, unlike other large-scale genomics cohorts that primarily focus on families and populations of European descent, our cohort originates from a diverse subpopulation that is poorly represented in public resources. Therefore, the baseline prevalence used in preprocessing might underestimate the true prevalence of the variants in this population. To overcome these difficulties and integrate the models’ predictions, we devised two ranking algorithms.

##### DSRank

DSRank considers the entire dataset’s scores distributions as a baseline to set a “benign” threshold. The normalized scores of the three ML models’ predictions are averaged over all available scores per variant. The individual model’s scores are normalized across our entire dataset using min-max normalization. Only variants that received scores from two or more ML models were included in this analysis (*n* = 1118). Variants with DSRank 2 SD or more above the calculated mean were prioritized for further analysis. As individual model scores were normalized over our dataset for each of the three scores separately, the final results are not affected by the pathogenic propensity of our dataset and the population cohort.

##### FAMRank

**Figure 3:**
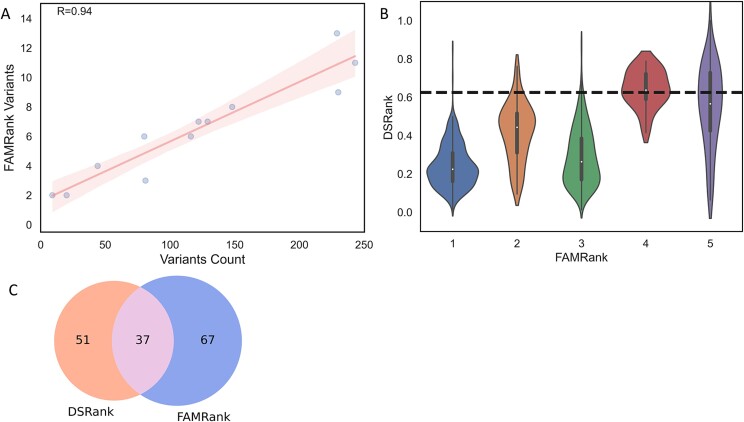
Ranking algorithms. (A) Number of variants per family (*x*-axis) versus number of variants with FAMRank 4–5 (*y*-axis), regression line (red) with 95% confidence interval (pink shade), *r*—Pearson correlation coefficient, *P*—two-tailed *P*-value. (B) DSRank distribution in relation to FAMRank, dotted line—DSRank threshold for pathogenic variants. (C) Pathogenic variants stratification by scoring function.

FAMRank exploits the underlying assumption that the participating families have a strong predisposition toward hereditary BC, due to the prevalence of BC and young age of diagnosis (Table 1). This suggests pathogenicity analysis should be conducted not only within the scope of the entire dataset but also within the subset of variants of each family. In essence, we try to find the most pathogenic variants within the subset of the family’s variants only, by using aggregative ranking algorithms. FAMRank provides a set of rules that classify each family-specific variant into five risk groups (Table 3). Variants were ranked as more likely to be pathogenic when multiple models ranked them within the top 10 percentile among the family’s subset of variants or they received a low DIST score or were prevalent in all family members or in multiple families. We assumed that FAMRank could uncover variants with slightly more subtle influence that would otherwise be overlooked by DSRank when matched against the most pathogenic variants of the entire cohort. Notably, FAMRank is affected by the number of variants per family (Fig. 3A) that may introduce noise when examining families with high variant counts. This effect can be mitigated by setting more stringent thresholds or by setting a hard cap adjusted to variant count per family. Interestingly, FAMRank and DSRank prioritized variants had marked overlap (Fig. 3C, Table 2) suggesting that the FAMRank signal-to-noise ratio is relatively high.

**Table 3:** Detailed description of variant scoring using FAMRank.

FAMRank	Conditions
5	Interface score below 4 Å **and** top 10 percentile by one or more models **or** Top 10 percentile by **all** three models **or** Recurring in all affected family members of two or more families^a^**and** top 10 percentile by one model in one of the families
4	Top 10 percentile by two models **or** Interface score below 4 Å
3	Top 10 percentile by one model **and** prevalent in all family members^a^
2	Top 10 percentile by one model
1	Otherwise

a Only for families with $n>1$ patients in study.

Variants’ scores were aggregated using the two ranking algorithms. If the variant was ranked above the pathogenicity threshold by either of the algorithms, it was prioritized for further analysis. DSRank classified 51 variants as candidate pathogenic, and FAMRank classified 67 variants as candidate pathogenic (top two risk groups). Overall, 81 unique candidate pathogenic variants from 80 genes across all 12 families were prioritized for further analysis. Although the ranking algorithms have marked overlap (37 variants), they also introduce unique variants that would not be otherwise prioritized (Fig. 3B).

#### Analysis

##### The candidate pathogenic variants are enriched with peroxisomal and mitochondrial proteins

**Figure 4:**
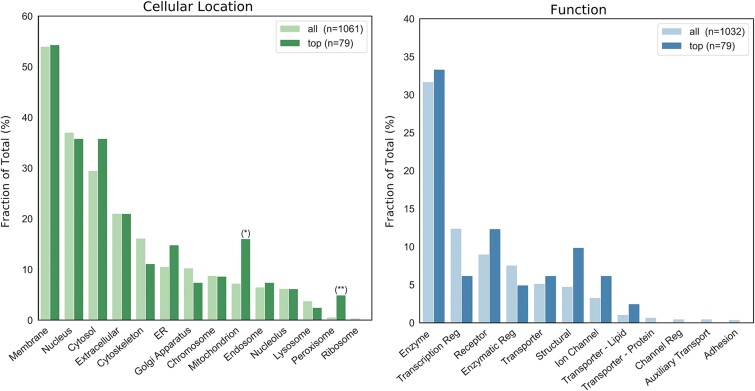
Enrichment analysis. (A) Cellular component and (B) Gene Ontology (GO) function annotations. *x*-axis; annotations, *y*-axis; ratio of annotated genes from subset, *n*—number of genes annotated with at least one of the annotations in *x*-axis. Note that candidate genes (dark colors) are a subset of all genes (light colors) (*)—significantly enriched (*P*-value = .004, FDR = 3.46e-2) (**)—significantly enriched (*P*-value = 2.50e-06, FDR = 3.75e-05).

**Figure 5:**
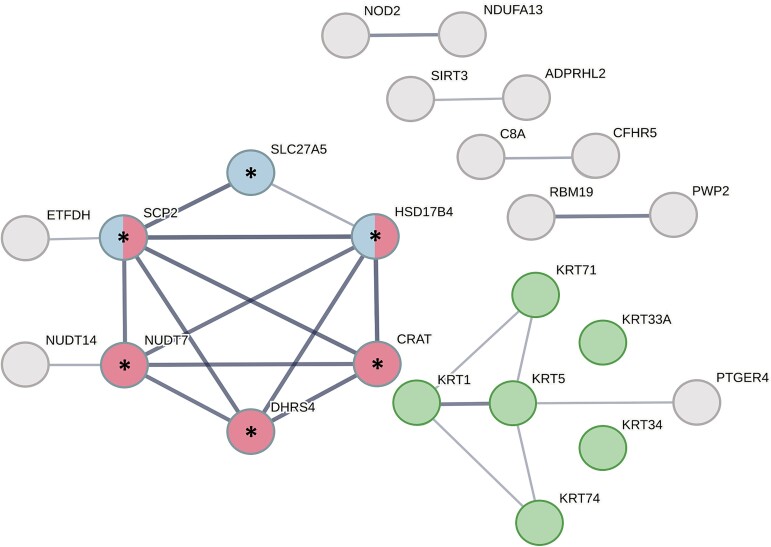
PPIs in the set of 80 proteins with candidate pathogenic variants. Colored nodes are selected functionally enriched networks: Peroxisomal matrix (red, FDR 0.0071), bile lipids metabolism (blue, FDR 0.01), and keratins (green, FDR 0.02). Edges width represents interaction confidence, disconnected nodes have no high-confidence interactions (interaction score > 0.5). Proteins without interactions are not shown for clarity, except keratin proteins. (*) represents a network with the highest confidence interactions (interaction score > 0.9).

To perform enrichment analysis, we assembled two sets of annotations from the Gene Ontology (GO) [37, 38] covering functional and cellular component aspects (Table S4 available online at http://bib.oxfordjournals.org/). We queried GoTermMapper [39] to compare GO annotations of the 80 candidate genes and the entire dataset (1123 genes). We found statistically significant enrichments of genes coding for peroxisomal and mitochondrial proteins (Fig. 4A). There was a trend for enrichment of structural proteins, mainly consisting of keratins that did not reach significance on multiple hypothesis correction (*P*-value = .04, Figs 4B and 5). Additionally, a trend in receptor proteins (*P*-value = .11) should also be noted. These trends might be proven significant given additional data.

In our analysis, we chose to focus on enrichment in the group of candidate pathogenic genes. Nonetheless, considering the cancer predisposition of the families in our cohort, it is also interesting to note annotations that achieved overall high ratios (Membrane, Nucleus, Cytosol, and Enzymes). These gene groups may have an important role in BC; however, none of them exhibit enrichment with PPIs (Fig. 5) nor in candidate pathogenic genes. Consequently, pinpointing genes of interest within these broader categories proved challenging.

##### The candidate pathogenic genes are significantly enriched with protein–protein interactions in common metabolic pathways recurring in multiple families

As an additional independent approach, we queried the 80 candidate genes using STRING [40], a database of functional PPIs. STRING assigns interaction scores in the range of [0–1] for each pair of proteins indicating the confidence of existing functional link between them. The interaction score is based on a combination of experimental evidence, co-expression data, text mining, genomic neighborhoods, gene fusions, and co-occurrences across different genomes.

Analysis of the candidate pathogenic genes showed significant PPI enrichment (*P*-values 2.86e-05 and 1.7e-06 for interaction score thresholds of 0.5 and 0.9, respectively) with a strong association to the peroxisomes and lipid metabolism [false discovery rate (FDR) 0.008] (Fig. 5). Eight genes containing candidate pathogenic variants in peroxisomal-related mechanisms recur across 7 of the 12 families in the study (SLC27A5, HSD17B4, CRAT, NUDT7, SCP2, DHRS4, ETFDH, NUDT14). Importantly six of the eight genes have the highest confidence interaction scores (>0.9).

Additionally, a network of keratins was also found to be marginally significant; however, they segregate only in two families and were not labeled with any significantly enriched GO annotation (Fig. 4). The rest of the interacting networks failed to show significant enrichment in any known pathway or cellular localization.

To account for the pathogenic propensity and population bias of our dataset, we performed additional validation by querying 20 randomly selected samples of 80 genes from our final dataset (*n* = 1123) in STRING. Using an interaction score setting of 0.5, none of the samples achieved a significant PPI enrichment score; the average *P*-value was .40 and standard deviation (SD) was 0.26. On an interaction score threshold of 0.9, none of the samples achieved a significant PPI enrichment score; the average *P*-value was 0.39 and SD 0.25.

##### Peroxisomal and lipid metabolism pathways are significantly correlated with breast cancer survival

**Figure 6:**
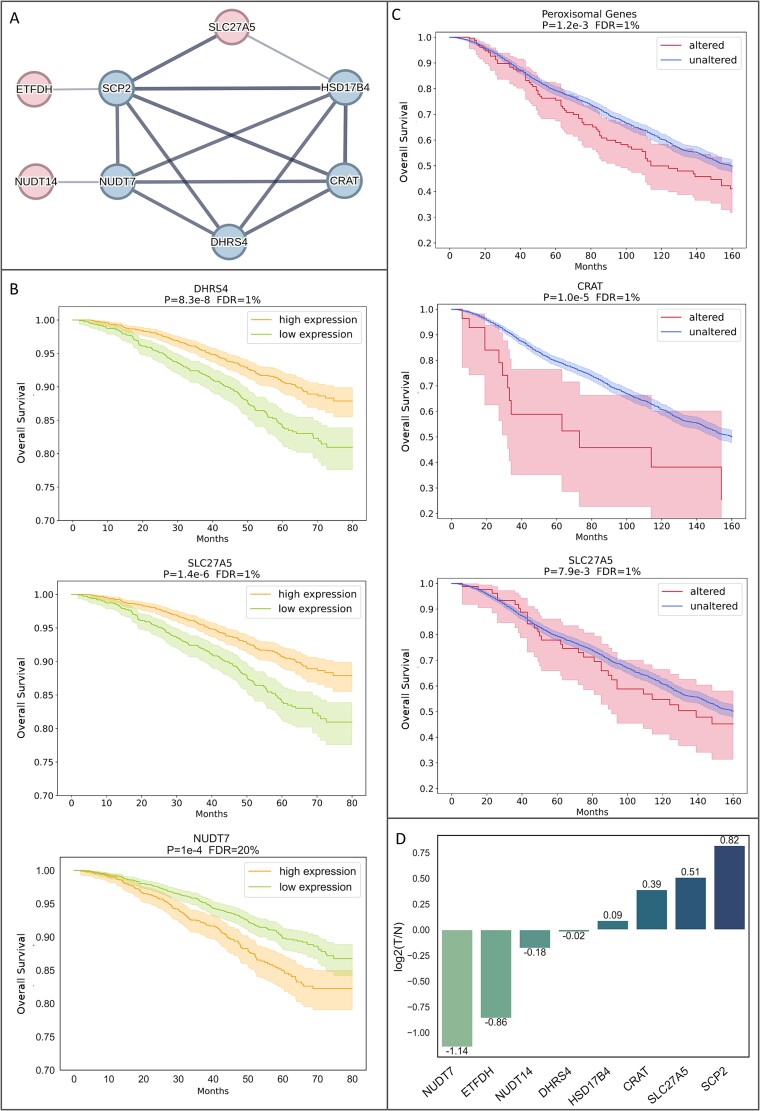
Effect of peroxisomal genes on breast cancer survival. (A) Peroxisomal genes tested in survival analysis. Colored nodes represent proteins in the peroxisomal matrix (blue) and bile lipids metabolism (red). Edges width represents interaction confidence. (B) Effect of gene expression (data obtained from KM-plotter) on BC survival. (C) Effect of somatic variants (data obtained from cBioPortal) in peroxisomal genes on breast cancer survival. (D) log2(Tumor/Normal) gene expression (canSAR). A value of |1| indicates a twofold expression difference. Patient count and events are shown in Table S5 available online at http://bib.oxfordjournals.org/. 95% confidence intervals are indicated by green, yellow, red, and blue shades.

To test whether the candidate pathogenic genes involved with peroxisomal and lipid metabolism pathways (Fig. 6A) are associated with BC, we tested publicly available datasets from BC patients. First, we analyzed the correlation between somatic pathogenic variants in these genes and BC survival. We queried all BC studies (*n* = 25) from cBioPortal [30, 31] and excluded overlapping samples. The query returned 11 632 samples from 10 851 patients. Seven percent of the queried patients had variants (either missense or loss of function) in one or more of the peroxisomal genes of the high confidence network (9% with genes of the relaxed network). The overall survival (OS) analysis (Fig. 6C) found two of the genes significantly associated with decreased survival when altered; CRAT (*P*-value 1.02e-5, FDR 2.55e-5, *n* = 29) and SLC27A5 (*P*-value 7.99e-3, FDR 0.01, *n* = 85) with median survival 73 and 94 months, respectively, compared to 156 months in control patients (*n*1 = 2817, *n*2 = 2825, respectively). The group of patients with alteration in at least one of the peroxisomal genes (high confidence network) also showed a significant decline in survival (*P*-value 1.19e-3, FDR 2.98e-3, *n* = 161) with a median survival of 111 months compared to 156 months in the control group. This pattern remains consistent even when examining only non-missense alterations in these genes (Fig. S1), indicating peroxisomal mechanisms may affect BC survival independently of missense mutations.

We then analyzed RNA-sequencing based on mRNA expression data using KM survival curves [29]. The BC dataset [29, 41] contains tumor samples from 2976 BC patients. We queried OS data with no restriction to specific tumor type, histologic features, or treatments and automatically selected the best cutoff for lower and upper expression groups. The survival analysis (Fig. 6B) revealed that for two of the genes, lower expression significantly decreased patient survival; DHRS4 (*P*-value 8.3e-8, FDR < 1%) and SLC27A5 (*P*-value 1.4e-6, FDR < 1%). As DHRS4 and SLC27A5 variants were identified in families 5 and 9, respectively, they could be validated by segregation in affected and unaffected family members. Importantly, both DHRS4 and SLC27A5 variants were not present in the unaffected family members. Other findings include NUDT7, which showed a reversed survival pattern, i.e. improved survival with lower expression. However, this association did not reach statistical significance on multiple hypotheses correction (*P*-value .0001, FDR < 20%).

We examined the differential gene expression of the peroxisomal gene collection in breast tumors versus healthy cells (Fig. 6D). For each of the genes, we extracted 1067 BC tumor samples from canSAR [42]. The log2(Tumor/Healthy) analysis showed SCP2 is overexpressed almost two-fold in tumor cells. A recent study demonstrated acquired SCP2:NTRK1 fusions associated with tumor progression on tamoxifen and letrozole treatment [43]. SCP2 was also involved in recurrent fusions with ECHDC2 and FAF1 in breast adenocarcinomas [44, 45]. These findings align with SCP2 overexpression and warrant further investigation. On the other hand, NUDT7 expression was decreased by over two-fold in tumor cells. Underexpression of NUDT7 seems to improve OS (Fig. 6B). In the context of germline pathogenic variants, this suggests that gain of function (GoF) variants in NUDT7 may contribute to BC predisposition and result in more aggressive tumors. Overall, we find that differential expression of these genes is associated with changes in OS.

### Structural analysis of CRAT and SLC27A5

To further investigate the effect of somatic pathogenic variants in CRAT and SLC27A5, we examined the mutational landscape at protein level by mapping all known missense variants from cBioPortal (Fig. S2).

**Figure 7:**
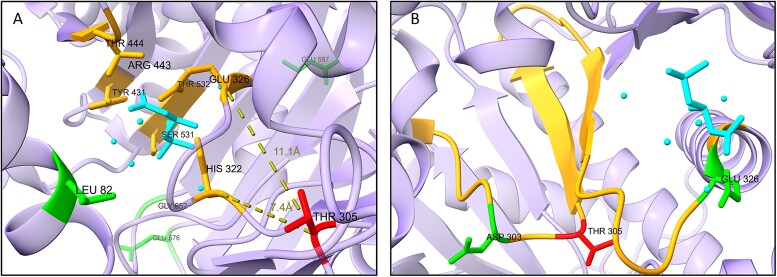
Structural analysis of CRAT variants (PDB 1S5O). The candidate pathogenic variant we identified (p.T305M) is in red (sticks), variants from cBioPortal are in green (sticks)—only variants in close proximity to the candidate pathogenic variant are presented, carnitine (and water molecules) are in cyan. (A) The amino acids that belong to the active catalytic site of carnitine are in orange. Glu326 also has variants in cBioPortal. Thr305 is 7.4 Å from His322 and 11.1 Å from Glu326. (B) A consensus sequence PS00440 (orange residues 299–327) is found in the catalytic tunnel of CRAT.

CRAT had 11 unique missense variants from nine different patients. Three altered amino acids (Leu82, Asp303, Glu326), were in close spatial proximity to our candidate pathogenic variant (p.T305M). These alterations are located in a consensus sequence found in the catalytic tunnel of the protein (Asp303, Tyr305, Glu326) and in close proximity to the carnitine binding site (Leu82, Tyr305, Glu326) [46, 47] (Fig. 7). Note that Glu326 is also directly involved in the carnitine catalytic site. Other variants (Gly484, Ala481) are found in close proximity to the coenzyme-A active site. SLC27A5 had 10 unique missense variants from 10 different patients. SLC27A5 structure is not well studied, and we were unable to find a specific pattern for these variants.

### DHRS4 potentially plays an underappreciated role in cancer

DHRS4 belongs to the short-chain dehydrogenases/reductases (SDRs) family and has dual localization to both the peroxisome and mitochondria [48, 49]. DHRS4 likely plays a role in lipid metabolism, but its exact *in vitro* function is unknown. However, it was shown to be an oncogene directly involved in the tumorigenicity of gliomas [50]. Our analysis shows underexpression of DHRS4 in BC is associated with poorer prognosis (Fig. 6B). Additionally, 2% of samples in cBioPortal (204/11 632 samples) had variants in the gene (Fig. S3). Furthermore, a healthy member (mother, age 79) did not exhibit any variants in the gene.

Our candidate pathogenic variant T84M is found on the β-sheets of the NADP/NAD-binding domain called the Rossmann fold [51]. However, it does not involve any known functional consensus sequences (Fig. S4A and B). By employing CSM-Potential2 [52], interface predictions were obtained for all residues of DHRS4 (PDB 3O4R), revealing that our identified variant is likely situated on a PPI interface (Fig. S4C and D), indicating a potential interference with PPIs. These findings advocate that DHRS4 might have an underappreciated role in cancer and warrant further investigation.

### Structural analysis of the identified variant in HSD17B4 suggests a loss-of-function mechanism

HSD17B4 is a gene encoding for 17β-HSD type 4, which is involved in peroxisomal beta-oxidation and in the degradation of 17β-estradiol into estrone [53]. Previous studies have shown that polymorphism in HSD17B1, which catalyzes similar steroidogenic reactions as HSD17B4, can be used to identify women at increased risk for advanced BC [54]. HSD17B4 was also mentioned as a potential therapeutic target in polycystic ovarian syndrome and BC [55]. More recent studies showed that pathological complete response in HER-2-positive BC to trastuzumab (HER2 inhibitor) plus chemotherapy can be predicted by HSD17B4 hypermethylation [56]. Additionally, loss of function (LoF) of HSD17B4 drives androgenesis in prostate cancer and is linked to the development of castration-resistant prostate cancer [57, 58]. HSD17B4 downregulation was also shown to be clinically significant in non-small-cell lung cancer [55, 59].

To support these findings, we queried publicly available *in vitro* CRISPR knockdown experiments from DEPMAP [60, 61]. HSD17B4 emerges as highly selective across multiple cancer cell lines and specifically BC (DepMap Public 23Q4 + Score, Chronos gene-affect score). This suggests that observed alterations in growth rates when knockdown are over 100 times more likely to have been sampled from a distribution of genes essential for cellular proliferation (skewed likelihood ratio > 100 times).

Furthermore, examining HSD17B4s’ top 25 co-dependent genes according to Chronos score in STRING, an extremely significant enrichment of pathways involving the peroxisome (FDR 6.81e-30) and peroxisome membrane (FDR 8.31e-28) emerges (Fig. S5), these also involve SCP2 (also within the candidate pathogenic genes list).

**Figure 8:**
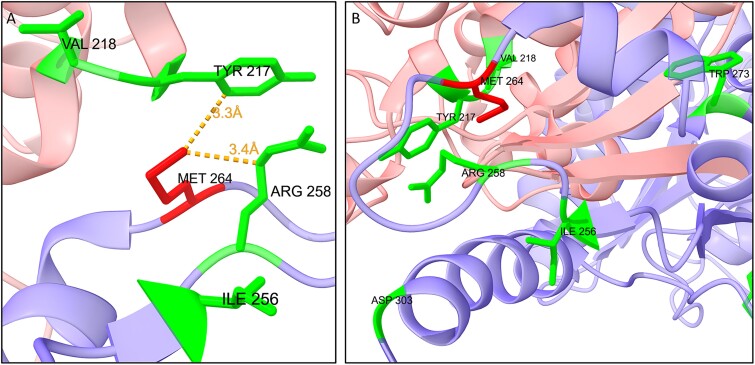
Structural analysis of HSD17B4. HSD17B4 functions as a dimer (pink and purple chains, PDB 1ZBQ). The variant found in our data (p.M124V) is shown in red (sticks), while residues in green (sticks) have known deleterious variants causing Perrault syndrome (Tyr217, Arg258, Asp303) and DBP. (A) Our identified variant is approximately 3 Å away from Arg258 and Tyr217. (B) Rotated view.

We also find that our candidate pathogenic variant (p.M124V) is found in close proximity to known deleterious variants in HSD17B4. Biallelic deleterious variants in this gene cause two different disorders: Perrault syndrome [62] and D-bifunctional protein deficiency (DBP) [32] (Fig. 8). DBP is a fatal disorder that causes neonatal hypotonia, seizures, hearing loss, and early death. Perrault syndrome is a rare disorder that causes progressive sensorineural hearing loss and ovarian dysgenesis in females. These findings suggest that our variant is likely to have a clinical effect on HSD17B4 function (LoF).

## Discussion

To date, the vast majority of familial BC cases cannot be attributed to variants in any specific gene [9]. While it is expected that additional BC predisposing genes are left to be discovered, outstanding high penetrance breast CPGs are likely to be extremely rare [7, 63]. Loveday *et al*. [7] recently published the largest white European, BRCA-negative whole-exome analysis to date (*n* = 2135). Their work excluded new high-penetrance breast CPGs using classical population analysis studies. The underlying assumption is that many of the pathogenic variants are rare and “family-specific” with moderate penetrance and variable phenotypes. Furthermore, the biological mechanism of these variants is likely to be polygenic and involve complex PPIs. Interpreting the pathogenicity of missense variants poses additional challenges, and classical methods are dependent on additional information such as recurrence in multiple patients [9]. Novel deep learning methods significantly extended our toolbox in quantifying the pathogenicity of missense variants using highly accurate predictors, such as EVE [15], ESM [13], and AlphaMissense [19].

Our work adapts these tools to the setting of family studies. We developed a novel approach at exploring germline missense variants; First, we analyze a unique cohort of 37 affected patients from 12 high-risk families of mixed Jewish origin. By analyzing rare variants that are shared among family members of non-European origin, we can study potentially rare, overlooked variants. Second, by employing multiple, highly accurate, deep learning–based approaches, we are able to give meaningful interpretation to the impact of missense variants at the protein level. The models’ predictions are independent of patients’ phenotype and prevalence in the general population. Third, our analysis is adapted to focus on high-risk families rather than individuals. Lastly, we conjointly analyze multiple variants to reveal common biological pathways rather than specific genes. Using our approach, we claim that CPGs can be used to reveal important mechanisms in cancer pathogenicity. Such mechanisms may be overlooked when examined in the context of the mutational landscape of tumors or when using classical population studies.

We have made the pipeline publicly available. Despite being validated on a small cohort of families, we believe it can provide clinicians with an automated tool to analyze similar familial data and draw meaningful conclusions. Application of the pipeline on comparable datasets will provide further validation. Nonetheless, we urge cautious interpretation of the pipeline’s findings.

Our work identified enrichment in peroxisomal and mitochondrial genes among the genes classified as candidate pathogenic by our model. While there has been a resurgence in appreciation of the importance of metabolic mechanisms in cancer initiation and progression [64], the role of peroxisomal and lipid metabolism pathways received little attention [65, 66]. In other species, the conservation of peroxisomal and bile lipid metabolism pathways was significantly associated with cancer resistance [67]. Peroxisomes are responsible for various metabolic pathways, including the processing of fatty acids and phospholipids. Moreover, a crosstalk between peroxisomes and mitochondria [23–28] including peroxisome–mitochondria interplay in ROS and calcium-sensing and regulation [23–25] further supports a potential role of peroxisomes in cancer. Defects in the peroxisomal pathway of bile acids biosynthesis lead to the accumulation of carcinogenic and toxic metabolites, enhance mitochondrial ROS production, and promote tumorigenesis [68–70]. Modeling the molecular development of BC in canine mammary tumors revealed that lipid metabolic processes were significantly enriched in BC progression expression patterns and in mammary carcinomas [71]. Additionally, recent evidence found enhanced expression of bile acid metabolism in a subset of breast and colon liver metastasis [72].

Further study of the peroxisomal candidate pathogenic genes identified in our study highlights that they are enriched with PPIs and share common pathways of lipid metabolism within the peroxisome matrix (Figs 4 and 5). Importantly, the peroxisome-related genes span across 7 out of 12 families in our study and cover diverse origins. This observation underscores mechanisms of the peroxisomal pathway in BC, highlighting its broader relevance beyond isolated familial occurrence. As additional supporting evidence, we examine the peroxisomal genes in the context of BC survival. The overlap between CPGs and cancer survival has been shown by various studies [73–76] including specific, well-established examples such as mismatch repair (MMR) genes in colorectal cancer [77, 78] and BRCA in ovarian cancer [79–81]. Variants in the peroxisomal genes recur in 9% of BC samples from cBioPortal. Furthermore, three of those genes were found to significantly affect BC survival when altered or underexpressed (Fig. 6). We finally narrowed our scope back to specific variants using protein structural analysis by examining our identified variants against established disease-driving pathogenic variants and variants documented in BC tumors. This analysis provides compelling evidence that specific variants in the genes CART and HSD17B4 are likely to interfere with protein function (Figs 7 and 8). Nonetheless, proving the clinical significance of specific missense variants will require additional gene perturbation experiments that are beyond the scope of this study and are left for future research.

Notably, several tumors exhibit alternation in peroxisome count and activity including colon [82], breast [83], and hepatocellular carcinoma [84]. In particular, a number of peroxisomal genes were shown to be related to BC tumorigenesis, survival, and response to treatment. The gene ACOX1 involved in beta oxidation was associated with poor prognosis in HER2-positive tumors when overexpressed [85]. In addition, downregulation of HSD17B4 was a reliable predictor of treatment success in HER-positive BC [86]. Interestingly ACOX1 and HSD17B4 exhibit strong functional links both in *Homo sapiens* and other organisms [40, 87–89]. Moreover, the gene AMACR, involved in pristanic acid breakdown, was overexpressed in BC [90] and other tumors. Furthermore, AGPS involved in ether lipids biosynthesis was shown to decrease BC tumorigenicity when inhibited [91].

From a clinical management perspective, optimal preventive strategies for most hereditary BC have not yet been determined. Many BC-associated CPGs play a role in DNA damage repair pathways and cell cycle checkpoint mechanisms. Understanding the functions of these genes can help accurately estimate the genetic risk of developing BC and guide the selection of appropriate preventive and therapeutic strategies for managing hereditary BC. We argue that expanding the catalog of hereditary predisposition to BC in high-risk women will significantly influence screening and follow-up recommendations. Nevertheless, identifying and validating new, rarely occurring predisposing genes can benefit from family studies that cover diverse origins and ethnic groups.

There are several important limitations to the study. First, our analysis focuses only on single-nucleotide missense variants, ignoring epigenetic landmarks and LoF variants that have a major effect on protein function. However, since missense variants are highly abundant in Next Generation Sequencing and understanding their impact poses a significant challenge we choose to focus our efforts there. Second, as our analysis is performed at the protein level, we are unable to investigate synonymous single-nucleotide variants (SNVs). These variants can have an impact at RNA and transcript levels but do not affect the analysis at the protein level. Third, we do not factor in patient’s clinical data such as penetrance within families, presenting age of patients, and environmental factors. Furthermore, we do not consider tumor types, hormonal status, and clinical outcomes in our final analysis. Fourth, we could not obtain the whole genome sequencing of tumor samples. Although current bioinformatic methods still struggle to separate driver from passenger variants [92], tumor samples could aid in shedding light on the biological mechanism and penetrance of the variants. Fifth, our analysis could also benefit from additional data on healthy family members (older, unaffected individuals). Currently, our dataset has only three healthy individuals. Nonetheless, as BC has an age-related phenotype with partial penetrance, incorporating data from healthy individuals is complex. Finally, our families are of non-European descent, which is poorly represented in public resources. Therefore, prevalence estimates may be inaccurate. Underestimation of SNVs’ prevalence may introduce noise (i.e. prevalent variants) to our analysis, while overestimation may result in overlooking significant variants.

## Conclusions

Here, we develop a novel framework for analyzing missense variants that are difficult to classify. We use state-of-the-art ML-based methods to focus on germline variants in BC. We further adapt our unique pipeline to familial cases. Our work suggests that peroxisomal mechanisms play an important, yet underappreciated, role in both germline BC predisposition and BC survival. We argue that understanding the role of peroxisomes in cancer metabolism represents a relatively unexplored area of research that could lead to new insights and therapeutic approaches.

## Materials and Methods

### Dataset and processing

We recruited BC patients from high-risk families. All participants had previously tested negative for cancer predisposition in standard gene panels [93]. High-risk families are defined as—at least three family members (first- and second-degree relatives) with BC, with at least one of them diagnosed with BC before age 50 (for pedigrees, see Fig. S6). The participants represent a population of patients from different Jewish Middle East, North Africa, and Ashkenazi origins. Genome data for all participants were collected by the Medical Genetics Institute in Shaare Zedek Medical Center (Jerusalem, Israel) between the years 2018 and 2019. DNA was extracted from whole blood samples using FlexiGene DNA kit (QIAGEN) according to the manufacturer’s protocol and sequenced on a DNBSEQ platform (BGI Genomics Co., Ltd., China). An average of 360 million 150 bp paired-end reads were generated per sample. Sequencing reads were aligned to the reference human genome (hg19) with BWA [94]. Alignment processing and variant calling were performed using GATK Variant Calling Best Practices guide [95].

We only include missense variants present in at least two affected family members and excluded variants with a prevalence (per variant) above 1% in the general population based on the updated general population compiled in gnomAD [36] (Table S2 available online at http://bib.oxfordjournals.org/, Table S8 available online at http://bib.oxfordjournals.org/). This threshold was chosen to exclude all SNPs from the study, defined as above 1% in the general population [96]. Additionally, we aimed to mitigate noise arising from frequency estimation biases that are not directly accounted for by our scoring models and are especially pronounced in under-represented populations such as those included in this study. These will be particularly challenging to handle given the normalization procedures applied across the entire dataset. This approach is further supported by diminished pathogenicity scores for variants with population frequency between 1% and 2% (*n* = 359): the ESM mean is −5.49 compared to −6.11 for 0–1%, *P*-value = 6.9e-4, the EVE mean is 0.23 compared to 0.18, *P*-value = 2.711e-6, and FIRM scores did not exhibit a significant difference.

### Protein metadata

For each protein in our dataset, we extracted amino acid sequences, isoform sequences, and entry names from the UniProtKB [97]. Queries were limited to human and reviewed genes only. In rare cases of unresolved discrepancy between the UniProtKB queried isoforms/entry names and those found in our dataset, we expanded the search to unreviewed (human) entries in an attempt to match the entries. One pseudogene (GLRA4) was excluded from our analysis. Available protein structures were extracted from the Protein Data Bank (PDB) [98].

### Variant pathogenicity scoring

Our variant scoring pipeline scores each variant using three separate ML-based pathogenicity prediction models and one structure-based algorithm (DIST) (Fig. 1). Approximately 92% of variants analyzed in the study were scored by two or more ML-based models, and 12% of variants were scored using the DIST algorithm (Table 4, Table S7 available online at http://bib.oxfordjournals.org/). Here, we provide technical details regarding the implementation and parameters used in our models and queries. Further description of the pipeline and models used is given in the Results section.

**Table 4:** Percentage of variants scored by each scoring model.

Model	% Variants covered (*N* = 1218)
EVEmodel	6% (*n* = 74)
EVEmodel + KNN imputation	82% (*n* = 1001)
ESM-v1	97% (*n* = 1182)
FIRM	73% (*n* = 891)
DIST	12% (*n* = 157)

#### EVEmodel

We queried EVE data published on November 2021 for all available proteins [15]. We used pathogenicity scores given by EVE class 75% set. Only a small fraction of all missense variants were directly covered in the EVE database (Table 4). For variants not covered directly by EVE, we used scores imputed from EVE using weighted *k*-nearest neighbors (KNN) imputation (CPT) [33]. EVE and CPT databases were queried directly to obtain pathogenicity scores. As there were some ambiguities among the protein sequences, we created a reference sequence of 10 amino acids surrounding the variant in question and searched for the reference sequence in EVE and CPT databases. The scores given by both models are in range 0 to 1 with 1 being the most pathogenic. Scores were normalized over the entire dataset using min-max normalization.

#### ESM-1v

To obtain a pathogenicity score for a variant based on a protein language model, we used the ESM-1v model (zero shot variant prediction - esm1v_t33_650M_UR90S) [13, 14]. We input a protein sequence with a [mask] token in the variant’s position within the protein sequence. ESM-1v only allows predictions for proteins smaller than 1022 amino acids. For longer proteins, we used a window of 1022 amino acids around the missense variant as the input protein sequence. We used the masked marginals score setting (“wt-marginals” strategy). The score is given using the log odds ratio at the mutated position between the mutant and the wild-type amino acid, given by:


(1) \begin{equation*} \log (\Pr \left({\mathrm{x}}_{\mathrm{i}}={\mathrm{AA}}_{\mathrm{mut}}\ |\ \mathrm{x}/{\mathrm{x}}_{\mathrm{i}}\right)-\log \left(\Pr \left({\mathrm{x}}_{\mathrm{i}}={\mathrm{AA}}_{\mathrm{wt}}\ |\ \mathrm{x}/{\mathrm{x}}_{\mathrm{i}}\right)\right) \end{equation*}

where ${x}_i$ denotes the [mask] location within the reference protein sequence and $A{A}_{mut}$ and $A{A}_{wt}$ denote the mutated and wild-type amino acids, respectively. $x/{x}_i$ denotes the reference sequence missing an amino acid at the [mask] position.

Masked marginal gives more negative scores to deleterious variants, but this score does not have a lower bound. To produce a score in the range [0,1] with 1 being the most deleterious, we use formula (2):


(2) \begin{equation*} 1-\operatorname{norm}\left(\mathrm{X}\right) \end{equation*}

where norm stands for min-max normalization and X for the set of ESM-1v scores over the entire dataset.

#### FIRM 

FIRM (Functional Impact Rating at the Molecular Level) is queried at the genome level [12]. We set FIRM using hg19 human genome and query using the alteration at DNA level. Scores are given between 0 and 1 with 1 being most pathogenic. The scores were normalized across the entire dataset using min-max normalization.

#### DIST score

This score was designed to assess the impact of the variant on PPIs. Variants found in close proximity to other chains are more likely to affect the inter-protein interaction. We measure the physical (${l}_2$) distance between all atoms of the variant amino acids and all the atoms of the adjacent chains in all PDB entries containing the variant in question. We run the search with incremental distance threshold values, specifically: 2, 3, 4, 6, 8, 12, and 16 Å. The DIST score is given by the lowest threshold that returns a non-empty list of amino acid pairs. Only variants with available structures in the PDB could be analyzed using this modality (Table 4).

#### Variant ranking

Variants’ scores were ranked using two ranking algorithms: DSRank and FAMRank (Results, Fig. 1). Variants that were ranked above a distinct threshold in either of the algorithms (candidate pathogenic variants) were prioritized for further analysis.

### Candidate pathogenic variants analysis

#### Gene Ontology

We test for enrichment of genes with candidate pathogenic variants in specific cellular components or with specific biological functions using annotations from the GO (Table S4 available online at http://bib.oxfordjournals.org/) [37, 38]. We queried GoTermMapper [39] to map both the entire dataset and the subset of candidate pathogenic proteins to the sets of annotations. Some of the genes were not labeled by any of the GO annotations and were therefore removed from this analysis. P-values were calculated using the Fisher’s exact test and corrected for multiple hypotheses using the Benjamini-Hochberg procedure. Only findings found significant after multiple hypothesis correction are reported in the study (FDR-corrected *P*-values <.05).

#### Protein–protein interaction enrichment

We queried STRING version 11.5 [40] using the set of candidate pathogenic variants’ proteins. Recently, a newer version of STRING (12.0) was released. The PPI networks were mostly similar in both versions with some variations in confidence score between interactions (Fig. S7, Table S6 available online at http://bib.oxfordjournals.org/). One notable difference is the highest confidence network (interaction score > 0.9) which was not significant in version 12.0 (*P*-value .088). PPI score computation is described in detail on the STRING website (https://string-db.org/cgi/help?sessionId=b8IMFunc3vTE).

#### Kaplan–Meier plotter

We queried KM-plotter breast RNA-seq data using the set of peroxisomal genes (Fig. 6A) [29]. We did not restrict our query to any specific tumor subtype or treatment regimen and used OS data. We split the patients into high-expression and low-expression groups using the automatic best cutoff selection. Overall, there were 2976 samples in the final survival analysis.

#### cBioPortal

We queried all available BC studies using the set of peroxisomal genes (Fig. 6A) [30, 31]. The query returned 11 632 samples from 10 851 patients. Survival analysis was done using OS data. Overlapping samples were excluded in the survival analyses.

#### Pipeline validation studies

Each stage of the pipeline was independently validated; we summarize here the various validations conducted throughout the study:

Scoring. ML models were all thoroughly validated on VEP benchmarks (Table S3 available online at http://bib.oxfordjournals.org/). We also show a high correlation between AlphaMissense scores and the scores of all ML models used in the pipeline as well as the aggregated DSRank (Fig. S8).

Ranking and analysis. Both DSRank and FAMRank integrate the various models’ scores to detect candidate pathogenic variants. Integration of VEP models to assess the pathogenicity of variants is supported by the work of Jagota *et al*. [33]. Furthermore, to demonstrate our ranking algorithms are capable of detecting credible signals within the data, we show that multiple (*n* = 20) random samples of 80 genes from the full dataset are not able to achieve any significant enrichment in STRING (see Results). Notably, this outcome is particularly impressive considering the preprocessing of the dataset, which excludes prevalent genes. Finally, enrichment analysis conducted using GO annotations demonstrated the candidate pathogenic variants significantly differed from the null (full dataset) distribution that would not be expected using random samples (Fig. 4).

**Key Points**

- We present a novel pipeline for the pathogenicity assessment of missense variants adapted for family studies using three complementary state-of-the-art machine-learning models and one structure-based algorithm.
- We apply our pipeline on a unique cohort of 12 high-risk families of Jewish Middle Eastern, North African, and Ashkenazi origins, which enables us to study potentially rare, overlooked variants.
- We further look beyond individual cancer-predisposing genes for enrichment in multiple genes and find that peroxisomal genes from common biological pathways recur among multiple families in our study and significantly affect breast cancer survival.
- Structural evidence shows the identified variants are found in close spatial proximity to other established deleterious variants.

## Supplementary Material

supplementary_figures_bbae346

## Data Availability

All variants reviewed in the study including family segregation are available in Table S8 available online at http://bib.oxfordjournals.org/. Final pathogenicity scores for all variants outputted by all models (following normalization) are available in Table S1 available online at http://bib.oxfordjournals.org/. All missense variants (without prevalence and family segregation preprocessing) are available at zenodo.org (10949088). Per special request please contact D.M.

## Software availability

Code for variant scoring and ranking using our pipeline, as well as a user friendly command line interface are available on our GitHub repository (https://github.com/gal-passi/FamAnalysis.git).

## Notes

**Gal Passi** is a PhD student at the school of Computer Science and a Medical Doctor, Hebrew University of Jerusalem. His research focuses on protein level analysis of human disease, integrating clinical data and deep learning.

**Sari Lieberman** is a PhD genetic counselor and a researcher in the Medical Genetics Institute at the Shaare Zedek Medical Center in Jerusalem. Her research focuses on the genetic basis of inherited cancer predisposition and its clinical implications.

**Fouad Zahdeh** is a bioinformatician specializing in short-read next-generation sequencing at Shaare Zedek Medical Center in Jerusalem. His research focuses on variant and copy number variation calling, with a keen interest in genome-wide association studies.

**Omer Murik** is a scientific researcher at the Institute of Medical Genetics, Shaare Zedek Medical Center in Jerusalem. His research focuses on identifying the genetic basis for inherited diseases, understanding the molecular mechanisms involved, and develops new omics-based tools to improve genetic testing of various disorders.

**Paul Renbaum** is the director of the Medical Genetics labs at the Shaare Zedek Medical Center in Jerusalem. His research focuses on identifying the genetic basis for inherited diseases and understanding the molecular mechanisms involved in Vrk1-related motor neuron disease.

**Rachel Beeri** is the director of the molecular diagnostic laboratory at the Shaare Zedek Medical Center in Jerusalem. She is involved in research projects mainly focused in identifying the genetic basis of inherited diseases.

**Michal Linial** is a professor at the Life Science Institute, Hebrew University of Jerusalem. Her research focuses on utilizing proteomics and genomics data to understand molecular mechanisms of complex diseases.

**Dalit May** is an MD/PhD senior physician specializing in family medicine and medical genetics. Her research focuses on the genetics and genomics of human development, physiology, and disease.

**Ephrat Levy-Lahad** is the Robin Chemers Neustein Director of the Medical Genetics Institute at Shaare Zedek Medical Center in Jerusalem, Israel and Professor of Internal Medicine and Medical Genetics at the Faculty of Medicine, the Hebrew University of Jerusalem. Her research focuses on the genetics of breast and ovarian cancer and on the genetic basis of rare diseases.

**Dina Schneidman-Duhovny** is a professor at the School of Computer Science and Engineering at the Hebrew University of Jerusalem. Her research focuses on structures of protein–protein interactions and macromolecular assemblies.
