# Supplementary material for: Discovering predisposing genes for hereditary breast cancer using deep learning
Source: Brief Bioinform. 2024 Jul 22;25(4):bbae346. doi: 10.1093/bib/bbae346 (PMC11262808; doi:10.1093/bib/bbae346)
Supplement: supplementary_figures_bbae346 [file supplementary_figures_bbae346.docx]

**Supplementary Figures**


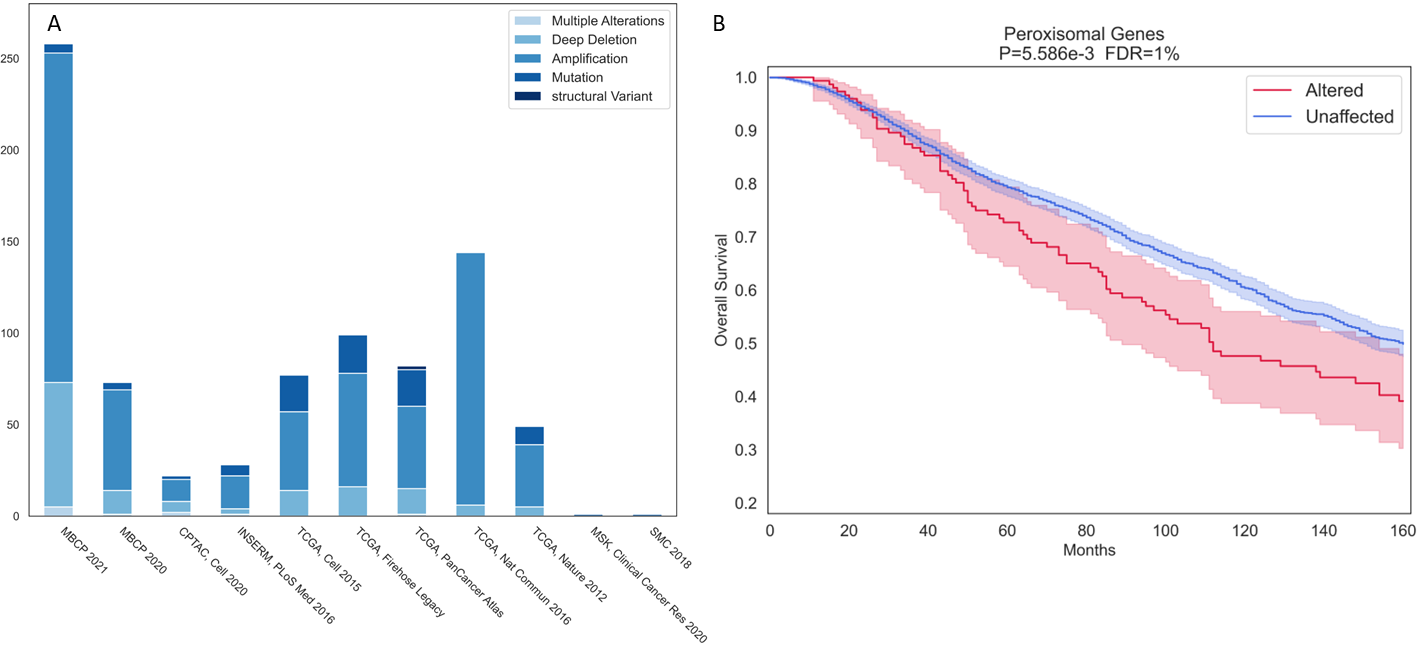


**Figure S1 Effect of non-missense alterations in peroxisomal genes on breast cancer survival** **A**. Distribution of genomic alteration in the peroxisomal genes across all studies included (n=12). MBCP - The Metastatic Breast Cancer Project, CPTAC - Proteogenomic landscape of breast cancer. **B**. Effect of non-missense variants (cBioPortal) in peroxisomal genes on breast cancer survival (Altered n=156, Unaffected n=2880, Events table available at Table S5), median survival - 111 months compared to 156 months in control HR -1.44.


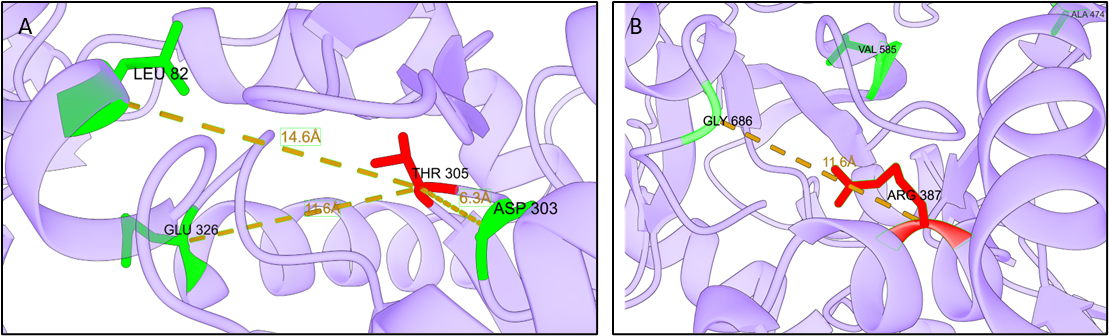


**Figure S2: CART and SLC27A5 Mutational landscape.** The candidate pathogenic variants we identified are shown in red (sticks), variants obtained from cBioPortal are shown in green (sticks) - only variants in close proximity to the candidate pathogenic variants are presented, distances are shown in yellow (angstrom).  **A** CART (PDB 1NM8) **B** SLC27A5 (AlphaFold - Q9Y2P5).


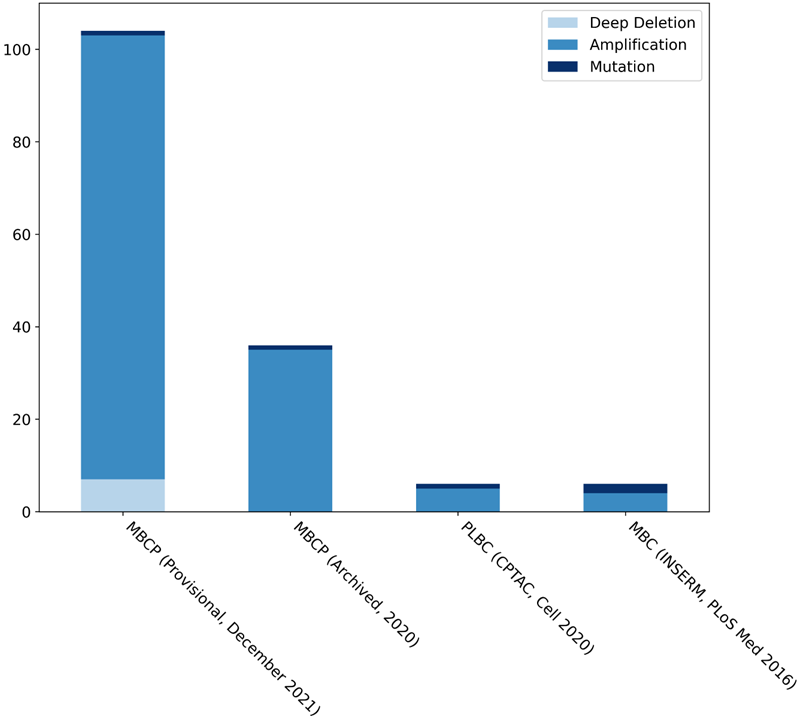


**Figure S3: DHRS4 somatic pathogenic variants** – studies from cBioPortal with at least 2% or and minimum of 20 samples per study including variants in DHRS4. MBCP - The Metastatic Breast Cancer Project, PLBC - Proteogenomic landscape of breast cancer, MBC - Metastatic Breast Cancer.

**
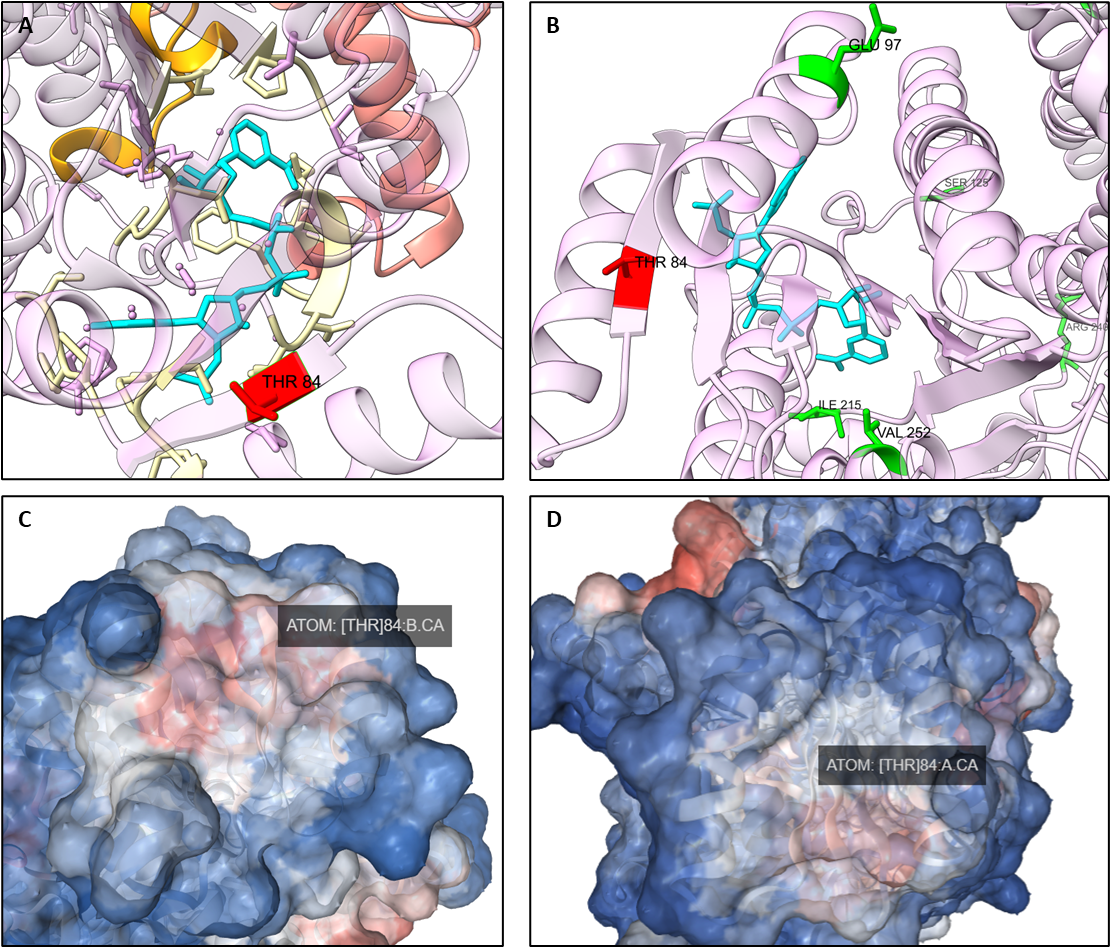
**

**Figure S4: DHRS4 structural analysis** (PDB 3O4R). Top figures; The candidate pathogenic variant we identified (T84M, red), NADP molecule (cyan). **A** Functional consensus motif (beige) [[54]](https://paperpile.com/c/yJ1iIj/tAyE); catalytic and coenzyme binding site (residues 41-44, 63-65, 88-90 ,116-118 ,167-169, 212-214, 217-220, orange) and active site (residues 170-181, 217-240, pink). **B** Reported variants from cBioPortal (residues 97,125, 215, 240, 252, green) only variants in close proximity to the candidate pathogenic variant are presented. Bottom figures interface prediction (PDB 3O4R) using CSM-Potential2; red indicates high interface score, blue indicates low interface score, scores range from 0 (low) to 1 (high). **C** view of identified variant in chain B (score 0.72). **D** view of identified variant in chain A (score 0.54).

**
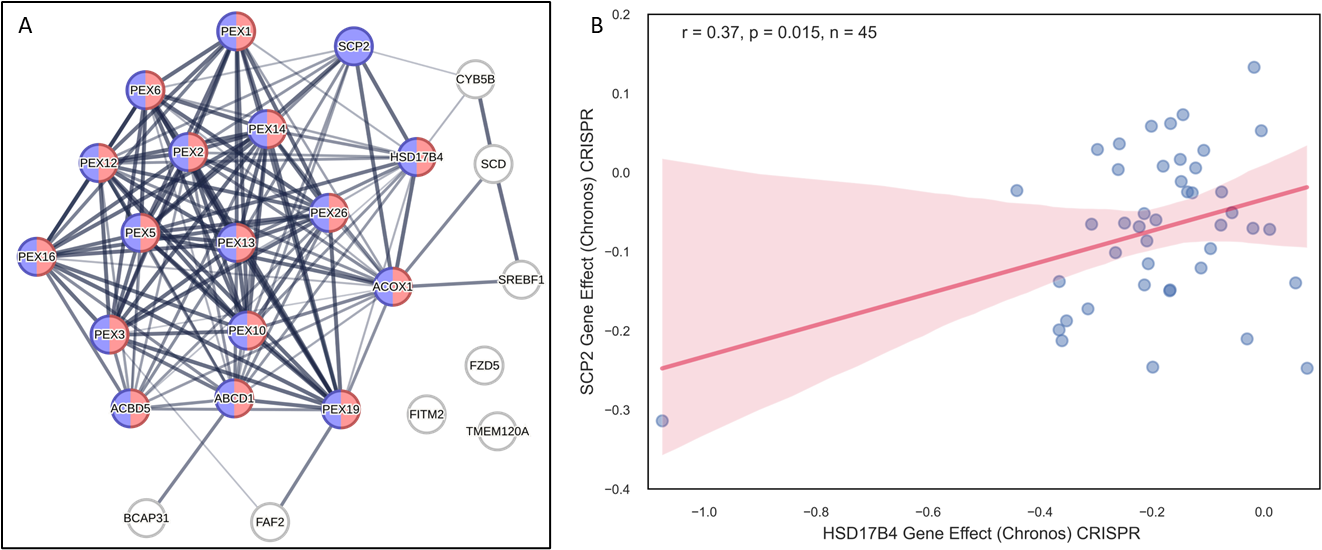
**

**Figure S5 HSD17B4 CRISPR in-vitro perturbation experiments** **A** PPI (STRING) of HSD17B4 top 25 co-dependent genes according to Chronos scores (DepMap Public 23Q4+Score, Chronos) . Colored nodes are selected functionally enriched networks: Peroxisomal membrane (red, FDR 6.81e-30) Peroxisome (blue, FDR 8.31e-28) **B** HSD17B4 and SCP2  Chronos gene-effect correlation in invasive breast carcinomas cell lineage  (DepMap Public 23Q4+Score, Chronos).


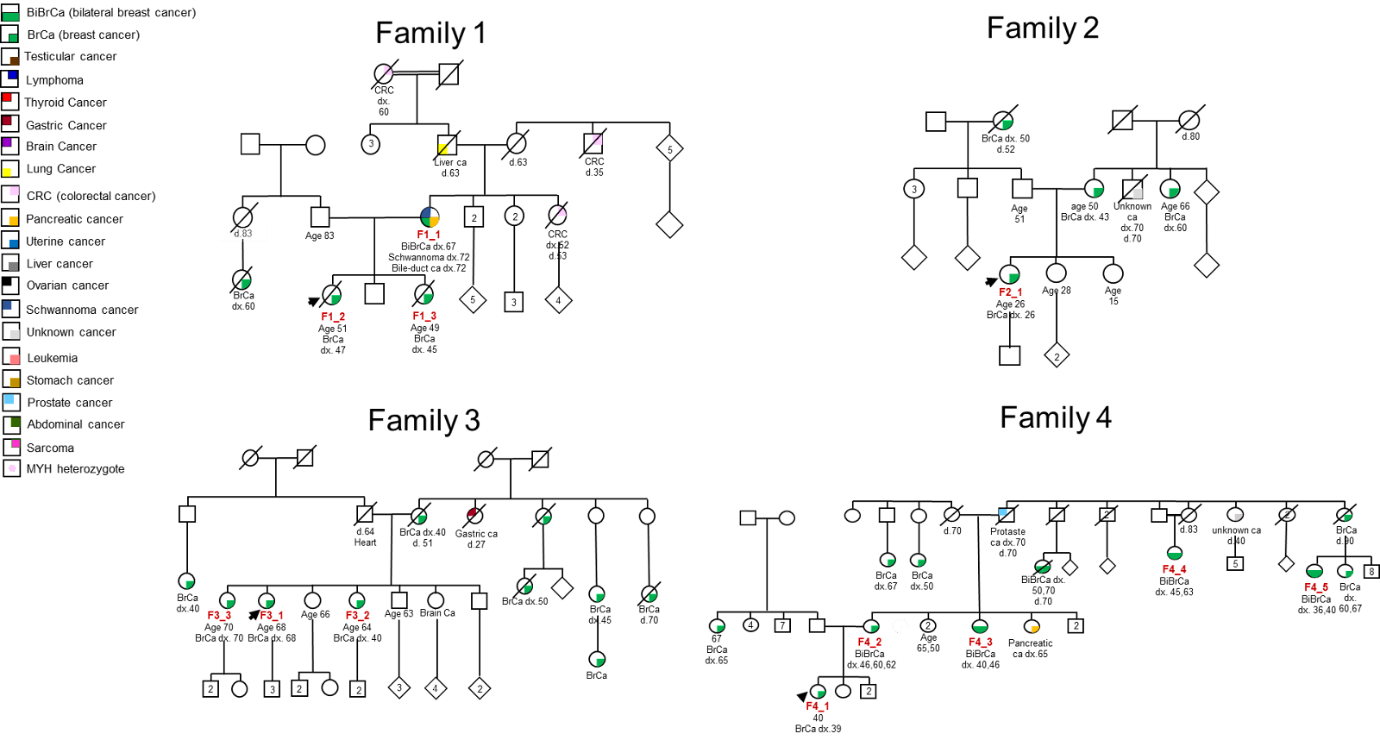


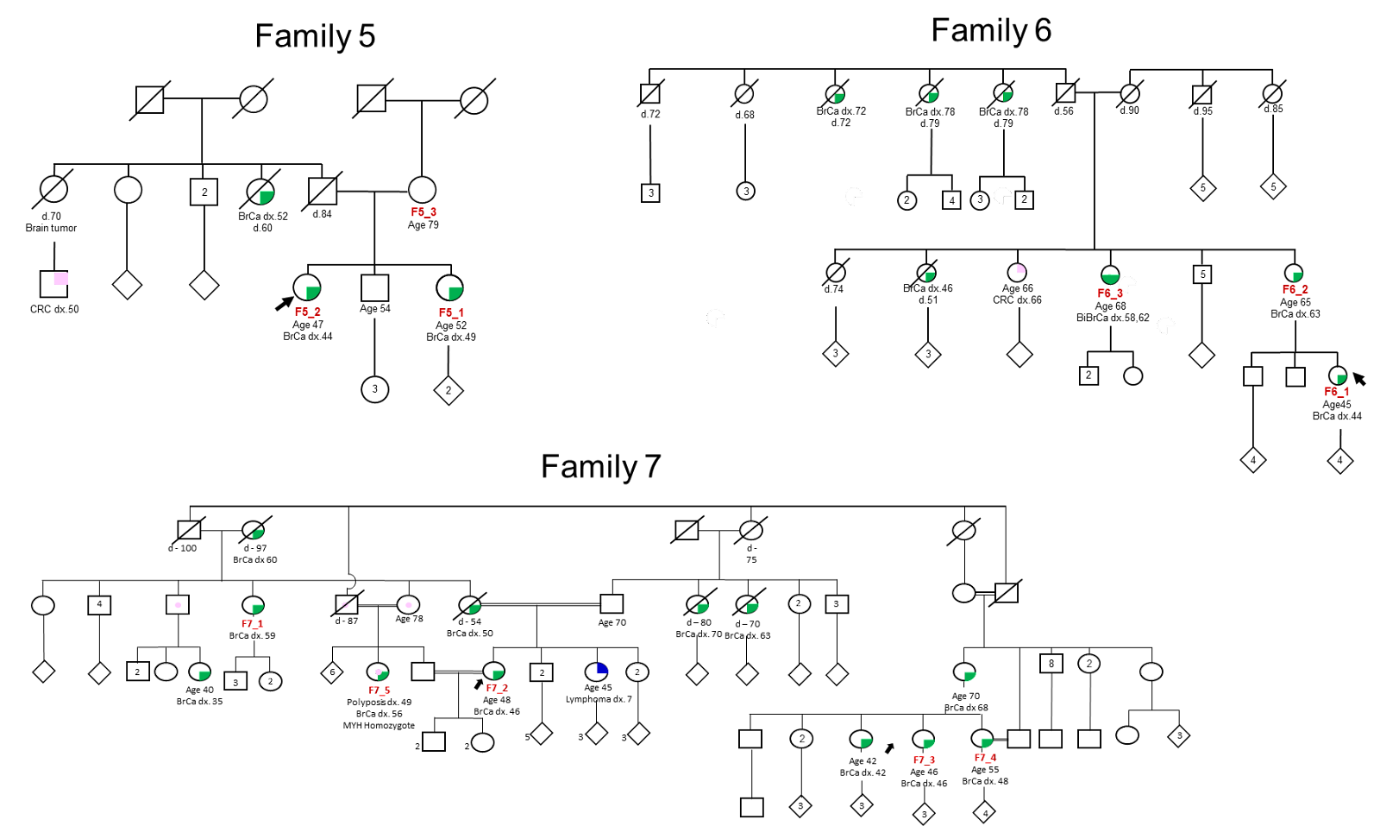


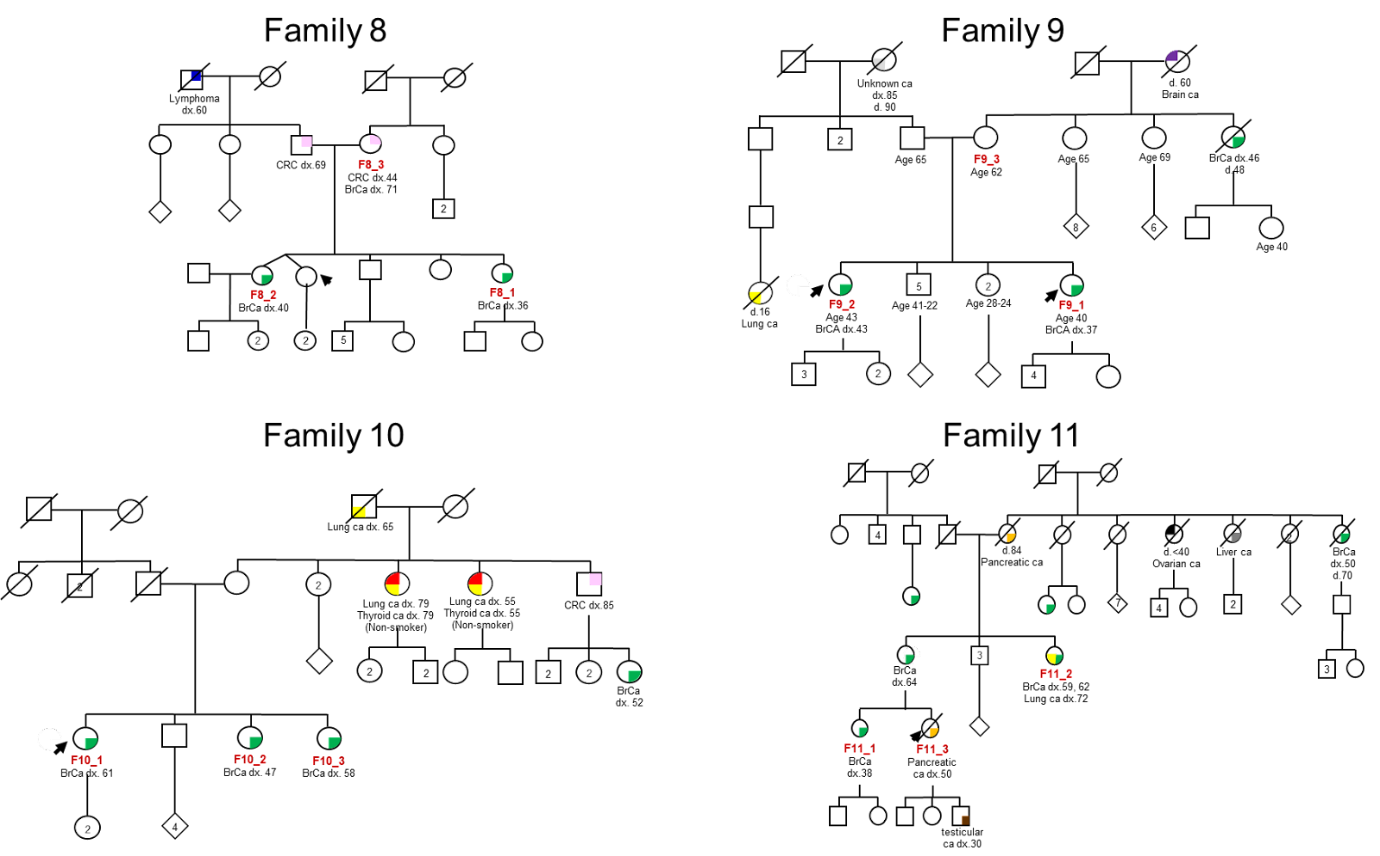

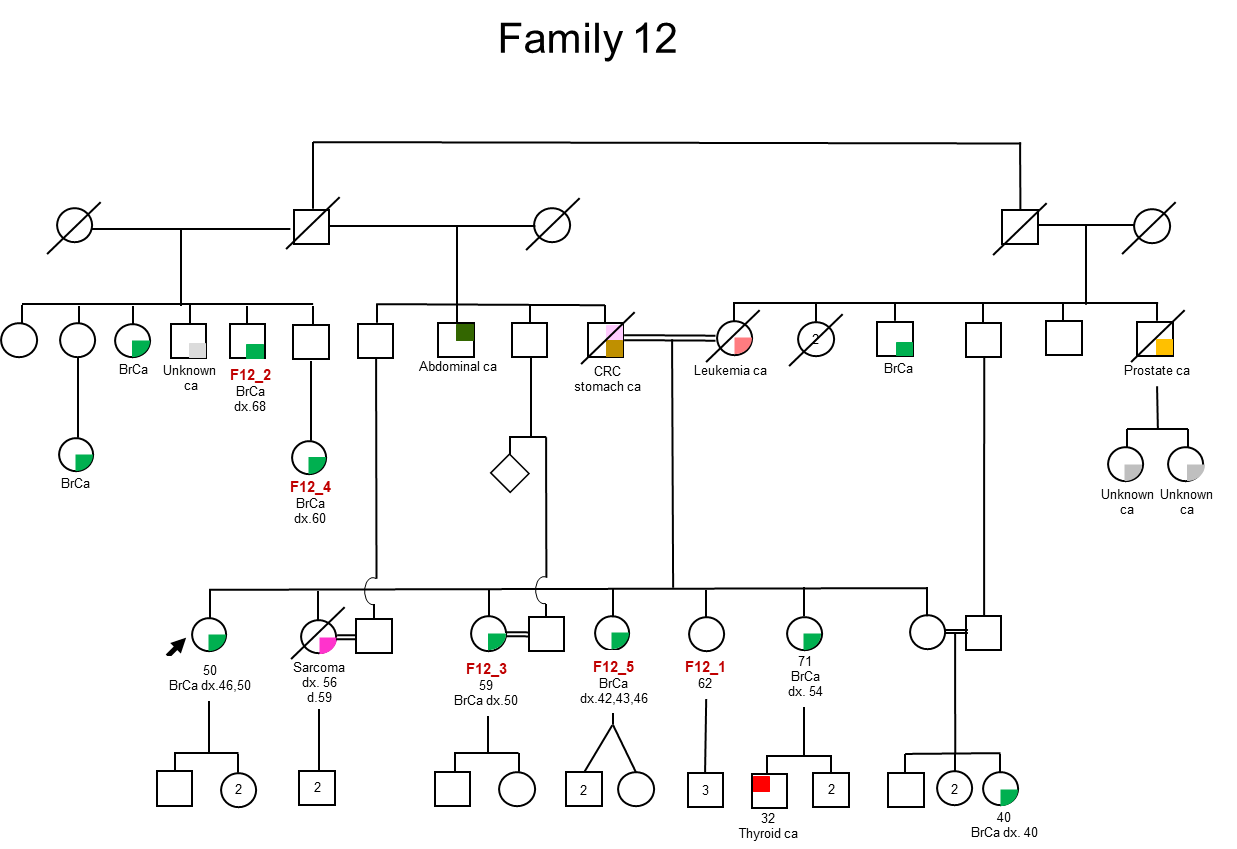


**Figure S6: Families Pedigrees** see legend (top left).


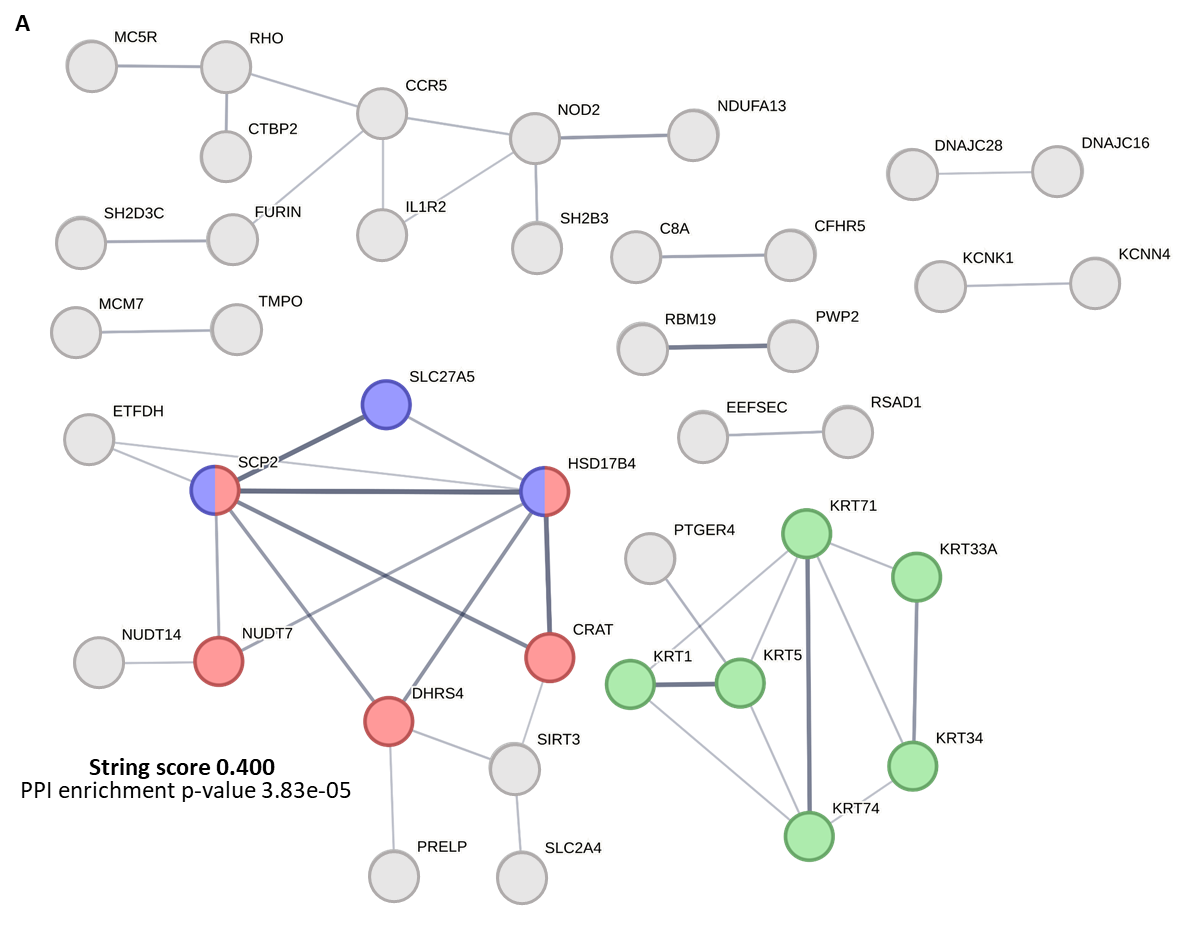


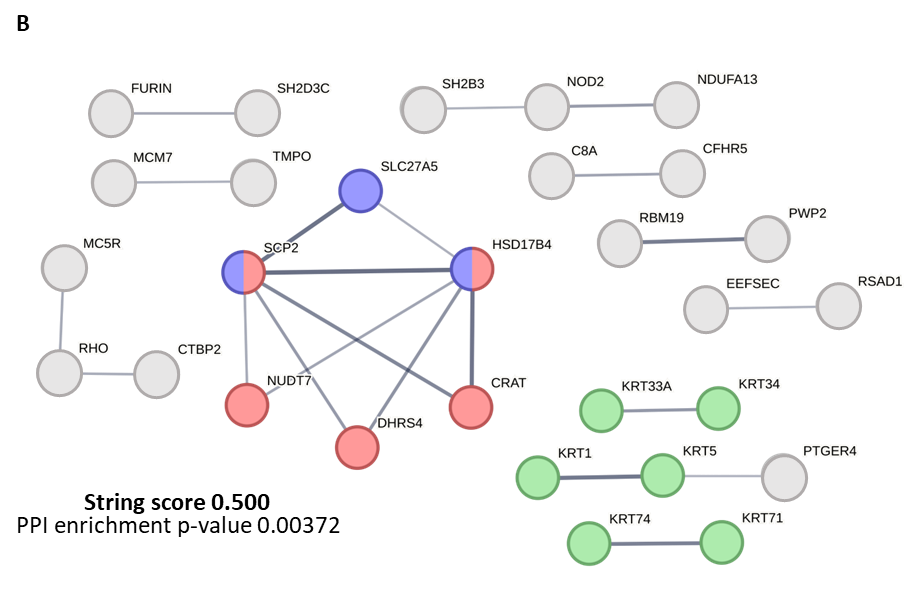


**Figure S7: STRING v12.0 PPI networks at interaction score thresholds** 0.4 (A) and 0.5 (B). Colored nodes are selected functionally enriched networks: peroxisomal matrix (red), bile lipids metabolism (blue), keratins (green). Edges width represent interaction confidence, disconnected nodes have no confidence interactions above the threshold. Proteins without interactions are not shown for clarity.

**
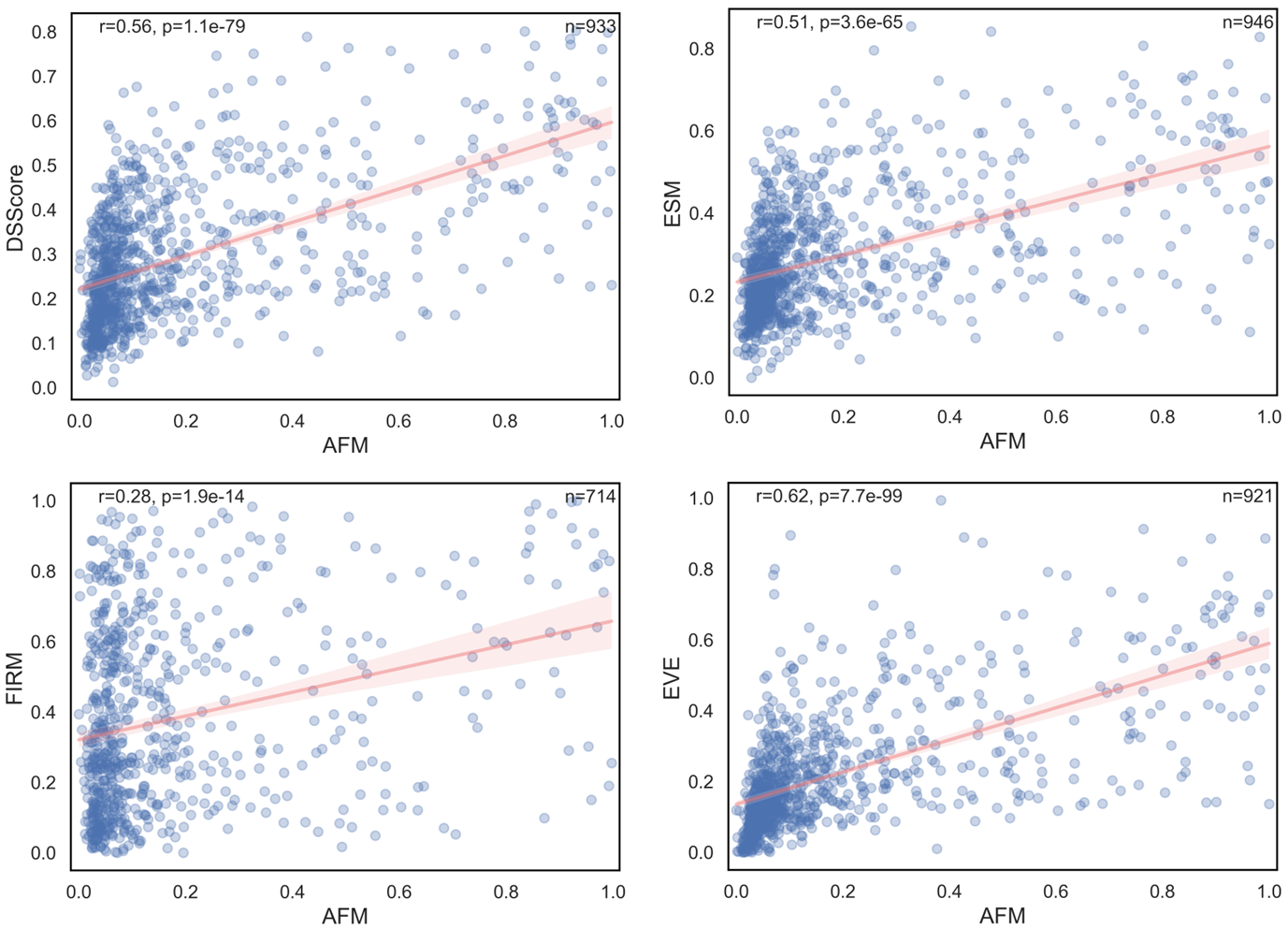
**

**Figure S8. Alpha Missense (AFM) correlation with our scores.** r – Pearson’s Correlation, p – p-value, n – number of samples. DSScore correlation was calculated only for variants which received scores from two or more models. AFM, ESM, FIRM, and EVE scores are normalized using min-max normalization.
